# Gestational testosterone excess early to mid-pregnancy disrupts maternal lipid homeostasis and activates biosynthesis of phosphoinositides and phosphatidylethanolamines in sheep

**DOI:** 10.1038/s41598-024-56886-6

**Published:** 2024-03-14

**Authors:** Nadia Saadat, Brooke Pallas, Joseph Ciarelli, Arpita Kalla Vyas, Vasantha Padmanabhan

**Affiliations:** 1 Department of Pediatrics, 7510 MSRB, University of Michigan, 1150 W. Medical Center Dr, Ann Arbor, MI 148019-5718 USA; 2 Unit Lab Animal Medicine, University of Michigan, Ann Arbor, MI USA; 3 Department of Pediatrics, Washington University St. Louis, St. Louis, MO USA

**Keywords:** Lipidome, Ovine, Pregnancy, Hyperandrogenism, Lipid markers, Developmental biology, Biomarkers, Endocrinology, Risk factors

## Abstract

Gestational hyperandrogenism is a risk factor for adverse maternal and offspring outcomes with effects likely mediated in part via disruptions in maternal lipid homeostasis. Using a translationally relevant sheep model of gestational testosterone (T) excess that manifests maternal hyperinsulinemia, intrauterine growth restriction (IUGR), and adverse offspring cardiometabolic outcomes, we tested if gestational T excess disrupts maternal lipidome. Dimensionality reduction models following shotgun lipidomics of gestational day 127.1 ± 5.3 (term 147 days) plasma revealed clear differences between control and T-treated sheep. Lipid signatures of gestational T-treated sheep included higher phosphoinositides (PI 36:2, 39:4) and lower acylcarnitines (CAR 16:0, 18:0, 18:1), phosphatidylcholines (PC 38:4, 40:5) and fatty acids (linoleic, arachidonic, Oleic). Gestational T excess activated phosphatidylethanolamines (PE) and PI biosynthesis. The reduction in key fatty acids may underlie IUGR and activated PI for the maternal hyperinsulinemia evidenced in this model. Maternal circulatory lipids contributing to adverse cardiometabolic outcomes are modifiable by dietary interventions.

## Introduction

Prenatal period is not only important for optimal development of the fetus but also in preparing how individuals respond to their environment for life after birth. Metabolic adaptations via changes in glucose, fatty acids and hormones occur to meet the demands of pregnancy, ensure sufficient energy stores, and provide optimal environment for optimal growth of fetus^1^. The routes of maternal communication with the fetus include endocrine, metabolic and immune signals^1^. Any compromise in this communication network will have detrimental impact on the developmental trajectory of the fetus resulting in poor birth outcomes and life-long health issues^1^. Prevailing evidence indicates that insults during pregnancy that lead to adverse consequences can stem from hormonal imbalances from disease states^2^, nutritional imbalances^3,4^, stress^5,6^ drugs^7–9^ and infectious agents^10–12^.

A hyperandrogenic state is one such insult^13–18^. Pregnant women are exposed inappropriately to excess androgens during critical periods of fetal organ differentiation through a variety of sources and disease states such as polycystic ovary syndrome (PCOS)^15–21^, congenital adrenal hyperplasia^22^, preeclampsia^23–25^ or exposure to endocrine disrupting chemicals that increase maternal androgen levels^26–28^. Our decades of research using sheep as a model system found that early- to mid-gestational testosterone (T) treatment virilizes the female fetus, leads to intrauterine growth restriction (IUGR), low birth weight, and metabolic and reproductive pathologies in the offspring^29,30^. Metabolic pathologies in the prenatal T-treated female offspring are manifested as peripheral and tissue-specific insulin resistance^31^, liver steatosis^32^, adipose defects^33–37^ and hypertension^38^. Reproductive pathologies are evidenced as irregular or absent estrous cycles^39,40^, multifollicular ovarian morphology^41^, follicular persistence^42^, and compromised fertility^43^. The bidirectional communication between the mother and fetus influenced predominantly by disruptions in the maternal milieu likely contributes to the observed adverse phenotypic outcomes in the offspring. For instance, evidence points to hyperandrogenic states such as PCOS being associated with perturbations in the maternal lipid^44^ and inflammatory milieu^45,46^. A thorough understanding of the biomolecular perturbations that occur in the maternal milieu in response to excess androgen exposure is therefore essential to identify mediators of adverse pregnancy and fetal outcomes to serve as biomarkers of adverse offspring outcomes and develop interventions.

In this regard, precocial animal models such as sheep which have a similar developmental trajectory as humans^47^ provide a valuable resource for understanding the impact of exposure to excess androgens on the maternal milieu and link to adverse metabolic perturbations occurring at the maternal level^48^. These include maternal hyperinsulinemia and decrease in acyl carnitines-fatty acid metabolites involved in many cellular energy metabolism pathways^48^.

Lipids, including acyl carnitines which are perturbed in the maternal milieu following gestational T treatment^48^, are essential metabolites that mediate several key cellular functions and fetal metabolic programming^49^. To ensure the health of the mother and meet the growth and developmental demands of the fetus, significant changes in lipid metabolism occur. As such, it is essential to expand upon our initial findings of a decrease in acyl carnitines^48^ to identify alterations in the multitude of lipids serving various key functions. Lipids fall under 8 major classes^50^. These include fatty acyls that include fatty acids; glycerolipids such as triglycerides that are essential for caloric storage; glycerophospholipids- structural lipids; sphingolipids such as ceramide involved in inflammation and infection; sterol lipids such as cholesterol that serve as steroid precursors hence key developmental programmers; prenol lipids that include carotenoids with antioxidant properties, saccharolipids such as lipid A, a component of lipopolysaccharide; and polyketides with immune-suppressing and anti-inflammatory activity.

Considering the key involvement of lipids in fetal growth and development and our initial finding pointing to an effect of testosterone excess on acyl carnitines, we undertook an untargeted lipidomics analysis of maternal plasma samples collected approximately a month after cessation of T treatment. We hypothesized gestational exposure to excess testosterone will perturb the maternal lipid homeostasis at several levels.

## Results

**Figure 1:**
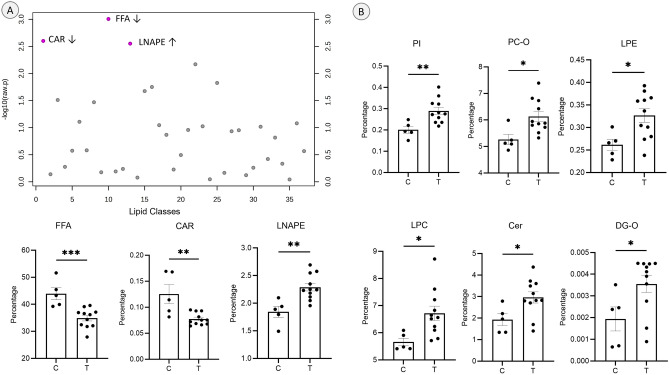
Differences in lipid classes after gestational T-treatment: Panel A: Scatter plot of all classes are shown on the top. The three lipid classes which were significant and met the FDR cutoff are shown in the bottom. Panel B: Other lipid classes which were significantly different between control and gestational T-treated sheep. Asterisks depict significance (* = *p* < 0.05, ** = *p* < 0.01, *** = *p* < 0.001).

Overall nine lipid classes showed differences in percentage of total lipids at the class level including free fatty acids (FFA), acylcarnitines (CAR), N-acyl lysophosphatidylethanolamine (LNAPE), phosphatidylinositols (PI), ether linked phosphatidylcholines (PC-O), lyso-phosphatidylethanolamines (LPE), lysophosphatidylcholines (LPC), ceramides (Cer) and ether linked diacylglycerols/diglycerides (DG-O) (*t*-test *p* < 0.05). Of these, FFA and CAR (showing lower levels) and LNAPE (showing higher levels) also met the FDR (false discovery rate) adjusted *t*-test *p* value criteria of < 0.05 (Fig. 1 left, panel A). Other classes showing differences including PI, PC-O, LPE, LPC, Cer and DG-O showed higher levels in the treated group compared to the controls (Fig. 1 right, panel B).

**Figure 2:**
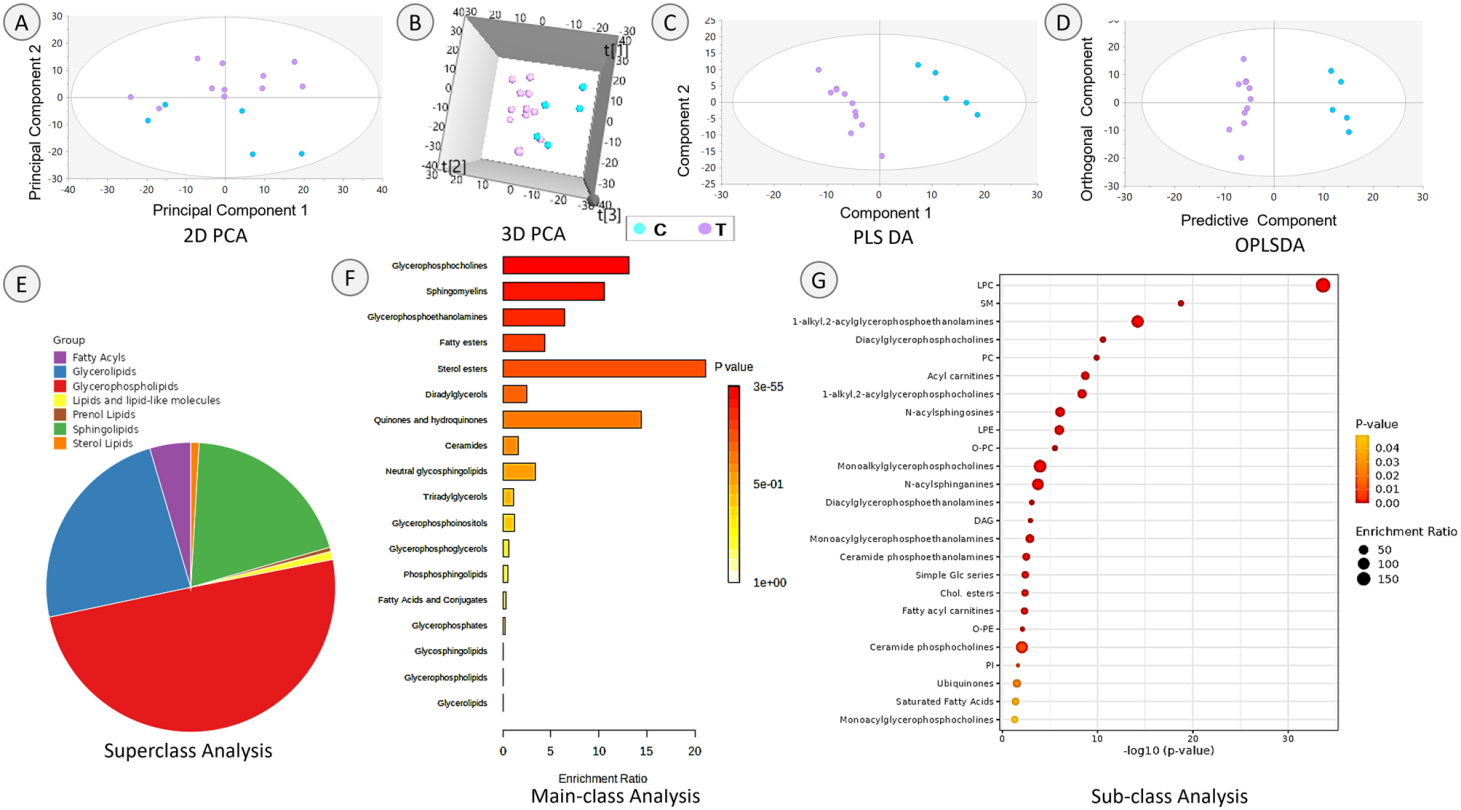
Effects of gestational T treatment on maternal lipidome profiles: Top: 2D PCA (**A**) and 3D PCA (**B**), PLS DA (**C**) and OPLS DA (**D**) score plots. For 2D and 3D PCA, principal component 1 is on *X*-axis and principal component 2 on *Y*-axis. PLS-DA plot is plotted with component 1 on *X*-axis and component 2 on *Y*-axis. OPLS DA is plotted with predictive component on *X*-axis and orthogonal component on *Y*-axis. Each dot represents one animal. Bottom: Lipid classes responsible for the sex-specific separation based on enrichment analysis. Left (**E**): pie chart representing contribution of Super-classes. Middle (**F**): enrichment ratio on the *X*-axis and the main classes on the *Y*-axis. Color gradients indicate significance range. Right (**G**): -log 10(*p* value) on *X*-axis and lipid Sub-classes on *Y*-axis. Color gradients indicate significance range with size of the dot indicating enrichment ratio.

Unsupervised PCA performed to get the overview of the lipidome data showed separation of the C and T groups in the 2D score plot, which was further improved in the 3D score plot indicative of distinct differences in lipidome profiles (Fig. 2, top, panels A and B). Supervised PLS DA and OPLS DA models validated this and showed distinct C and T groupings (Fig. 2, top panels C and D). Lipid species with higher VIP values above 1 indicating importance to the model for OPLS DA model were used for the enrichment analysis. The majority of the lipids enriched at the super-class level were glycerophospholipids, sphingolipids and glycerolipids (Fig. 2 bottom, panel E). The lipids with high enrichment ratio in the main classes included sterol esters, glycerophosphocholines, sphingomyelins (SM) and glycerophosphoethanolamines, substantiating the contribution of these lipid classes for the differences in lipidome profiles between C and T groups. Quinones and hydroquinones had high enrichment ratio but lower statistical significance (Fig. 2, bottom, panel F). At the sub-class level LPC, SM, 1-alkyl, 2-acylglycerophosphoethanolamines, diacylglycerophosphocholines, PC, acyl carnitines, 1 alkyl-2 acylglycerophosphocholines, N-acylsphingosines, LPE, ether linked PC, monoalkylglycerophosphocholines, N-acylsphinganines were among the top lipids based on statistical significance. Among these LPC, 1-alkyl, 2-acylglycerophosphoethanolamines, acyl carnitines, 1 alkyl-2acylglycerophosphocholines, N-acylsphingosines, LPE, monoalkylglycerophosphocholines and N-acylsphinganines had enrichment ratio above 100 (Fig. 2, bottom, panel G).

**Figure 3:**
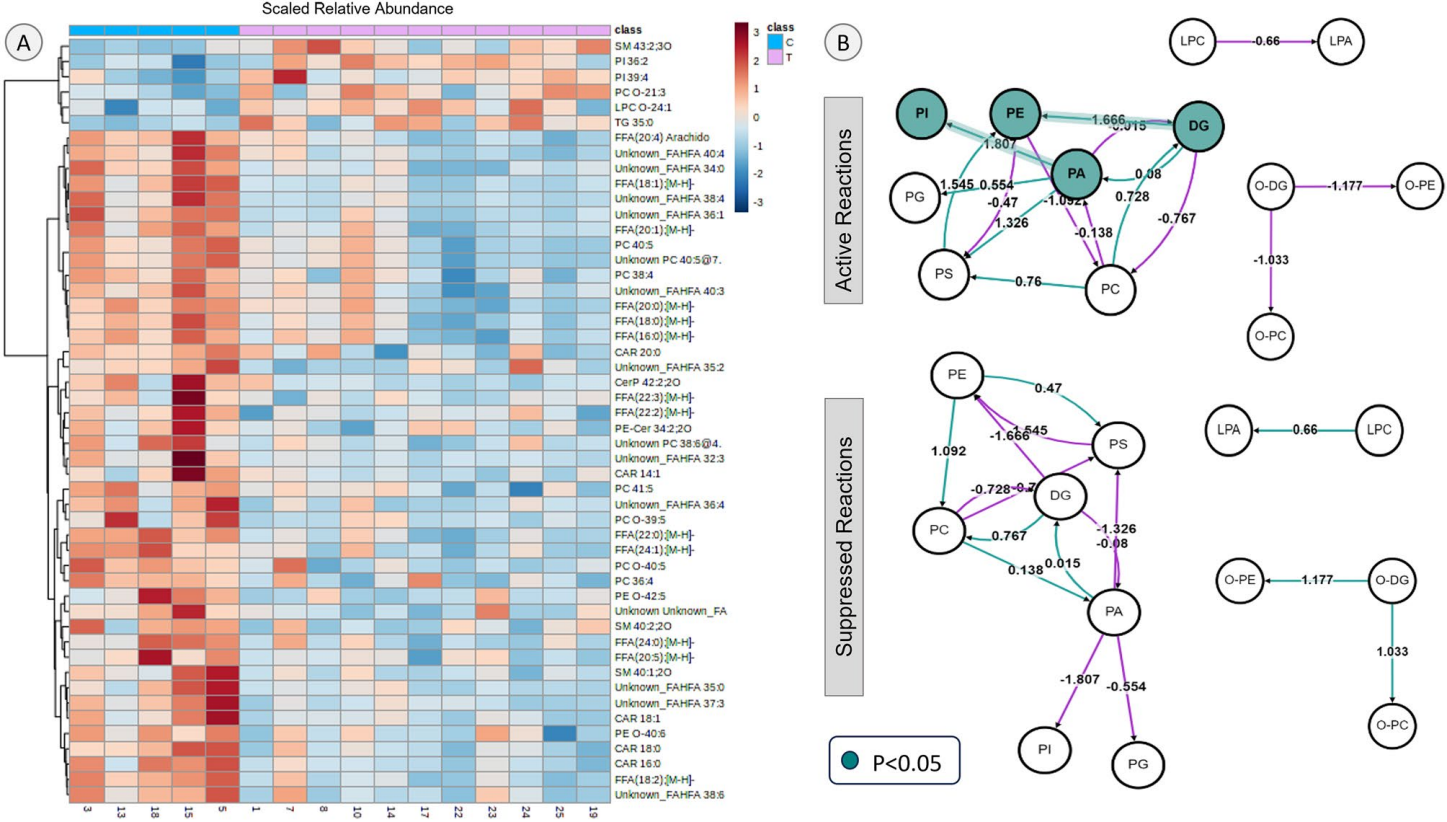
Lipid species and metabolic reactions affected by T-treatment during pregnancy: Panel A: Heatmap showing top 50 scaled relative abundance of lipid species based on student’s t distribution. Red represents higher levels and blue represents lower levels. Panel B: Active and suppressed reactions in T-treated compared to control pregnant ewes. Green circles represent significant reactions. Z score above 1.645 *p* < 0.05 represent significance.

Top lipid species and lipid metabolism pathways perturbed by gestational testosterone excess: The top 50 lipid species accounting for differences in lipidome profiles based on student’s t distribution are shown in Heatmap (Fig. 3, left, panel A), the t-statistic, *t*-test *p* value and FDR adjusted *p*- values for top 50 lipid species are shown in Supplementary Table 1. Among these top 50 species, only few lipids including some phosphoinositide species- PI 36:2, 39:4, sphingomyelin SM 43:2;3O, ether linked PC- PC-O-21:3, ether linked LPC- LPC O-24:1 and TG 35:0 were found to be higher in the T group compared to controls. In contrast, a large number of lipid species were present in lower levels in the T group compared to controls. Among the lower ones were key unsaturated FFA, linoleic acid (18:2), arachidonic acid (20:4), oleic acid (18:1), unsaturated FFA 20:1, 22:3, 22:2, 24:1, 20:5, 18:2, and saturated FFA- 20:0, 16:0, 22:0, 24:0. Several acylcarnitine species including CAR 20:0, 14:1, 18:1, 18:0, 16:0, were lower in the T-treated group. Some phosphatidylcholine species were also in lower concentration in the T group, namely PC 40:5, 38:4, 41:5, 36:4 and ether linked PC species PC-O 39:5 and 40:5. Additionally, many unknown lipid species specifically unknown fatty acid esters of hydroxy fatty acids (FAHFA) species were present in lower levels in the T group compared to C group (Fig. 3 left, panel A).

Pathway analysis performed with BioPAN on LIPID MAPS platform found at the subclass level, conversion of PE from DG (z-score = 1.666, *p* < 0.05) and conversion of PI from phosphatidic acid (z-score = 1.807, *p* < 0.05) to be the activated reactions in the T group compared to controls (Fig. 3, panel B, top right). Barring marginal suppression of biosynthesis of PC from PE (z-score = 1.092) (Fig. 3, panel B, bottom right), no significant suppressed reactions were evident. Significant lipid species level active and suppressed pathways of relevance, along with pathway classification, are listed in Supplementary Table 2. Cumulative level analyses found the biosynthesis PE from DG was the activated sub class level pathway (cumulative z score of 1.66, *p* < 0.05). All significant lipid species active reactions are listed in Supplementary Table 3 and suppressed reactions in Supplementary Table 4.

**Figure 4:**
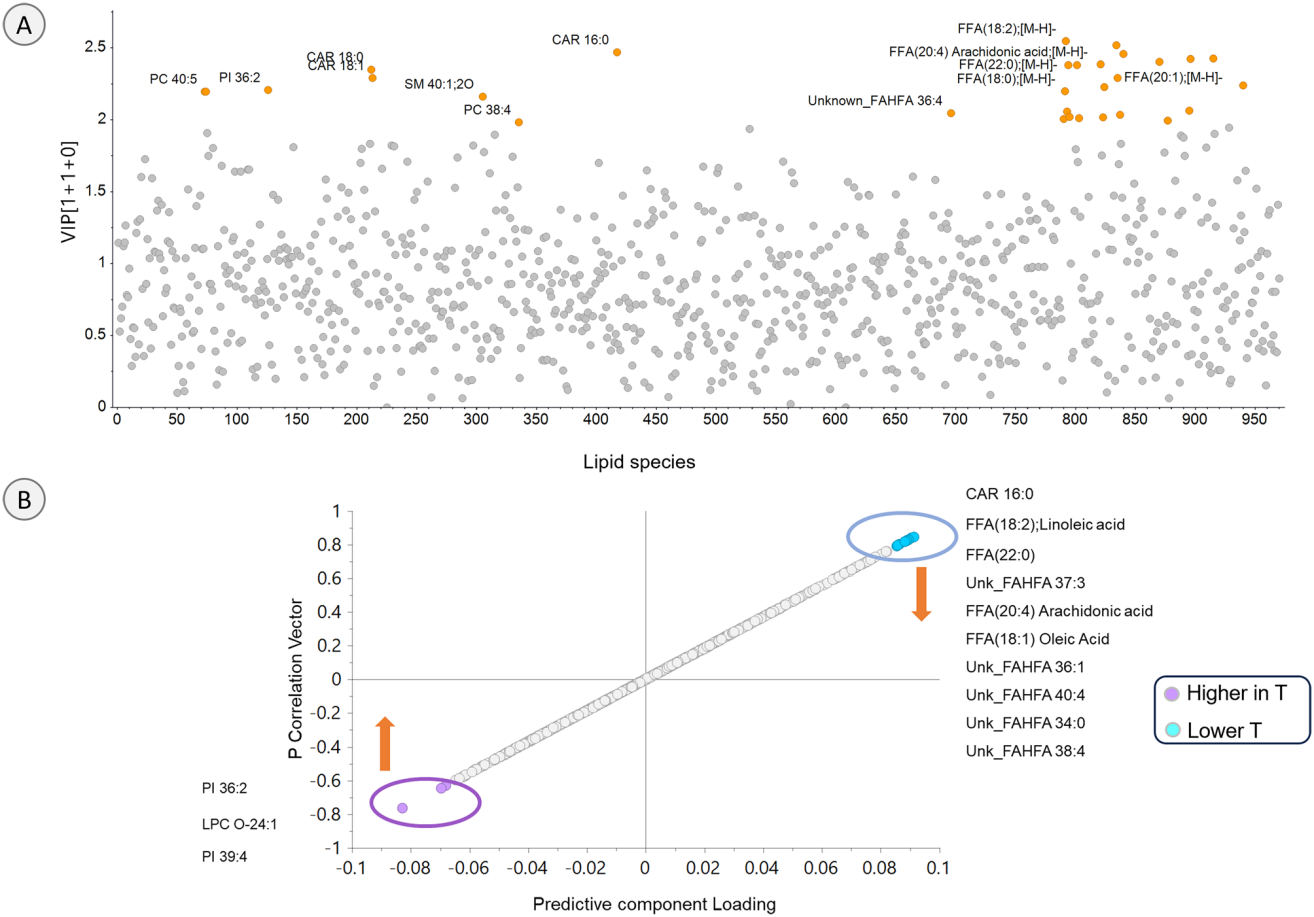
Potential signatures of gestational T-treated sheep: Panel A: OPLS DA VIP plot showing lipid signatures with high VIP values in orange. Panel B: OPLS DA S-plot with potential biomarkers of T treatment. Purple represents higher and light blue represents lower levels in T-treated ewes. Each point on S-plot represents individual lipid species.

Potential lipid signatures of gestational T excess: VIP plot (Fig. 4, top panel A) and S-plot (Fig. 4 bottom, panel B) from the OPLS DA multivariate model were used to identify the potential lipid biomarkers of gestational T treatment. Higher VIP value of the lipids are more relevant to the multivariate model and responsible for the separation of two groups in the OPLS DA score plot. The selected lipids with higher VIP values are labeled and colored orange in the VIP plot (Fig. 4, top, panel A). The identified potential lipid signatures along with VIP values are listed in Table 1.

**Table 1:** Potential Lipid signatures of excess T treatment.

Lipid Species	VIP values
PC 40:5	2.19501
Unknown PC 40: 5@7.38	2.19501
PI 36:2	2.20725
CAR 18:0	2.34932
CAR 18:1	2.29046
SM 40:1;2O	2.16227
PC 38:4	1.98383
CAR 16:0	2.46837
Unknown PC 38: 6@4.72	1.93587
Unknown_FAHFA 36:4	2.04743
FFA (16:0)	2.00447
FFA (18:0)	2.19848
FFA (18:2)	2.54704
FFA (20:0)	2.05644
FFA (22:0)-	2.38108
FFA (24:0)	2.02
Unknown_FAHFA 37:3	2.37958
Unknown_FAHFA 40:3	2.01014
FFA (20:4)	2.38678
CerP 42:2;2O	2.01651
Unknown_FAHFA 35:0	2.22794
FFA (18:1)	2.51922
FFA (20:1)	2.29104
FFA (24:1)	2.03395
Unknown_FAHFA 36:1	2.45766
Unknown_FAHFA 40:4	2.40434
PC O-40:5	1.99348
Unknown_FAHFA 32:3	2.06427
Unknown_FAHFA 34:0	2.42309
Unknown_FAHFA 38:4	2.42614

Specific signatures with high magnitude and significance were identified using S-plot (Fig. 4, bottom, panel B). The lipids on the bottom left side of the S-plot representing lipids which were found to be higher in T group compared to C group included three lipid species, PI 36:2 and 39:4, and LPC O-24:1. On the other hand the lipid species at top right corner represents lipid markers lower in the T group which included, L-palmitoylcarnitine (CAR 16:0), and unsaturated FFA (linoleic acid, 18:2; arachidonic, 20:4; Oleic,18:1) and saturated FFA 22:0. Several unknown lipids belonging to FAHFA species were also found to be at lower levels in the T treated group compared to C group. The significance of all the potential lipid biomarkers identified by the S-plot were confirmed by the confidence interval of loadings.

Retrospective power analysis of the lipid signatures found all the potential signatures to have good power (with alpha set at 0.05) with 11 T animals and 5 controls. Power around 0.8 (80% chance to detect statistically significant differences) and above is considered good acceptable power to reject the null hypothesis. The power of lipid markers that are higher in the T-treated group included, PI 36:2 = 0.936 and PI 39:4 = 0.800, and LPC O-24:1 = 0.786. The lipid markers that are lower in the T-treated group included CAR 16:0 = 1, PC 38:4 = 0.812, unsaturated FFA -linoleic acid, 18:2 = 0.998; arachidonic, 20:4 = 0.995; Oleic,18:1 = 1 and saturated FFA 22:0 = 0.998.

**Figure 5:**
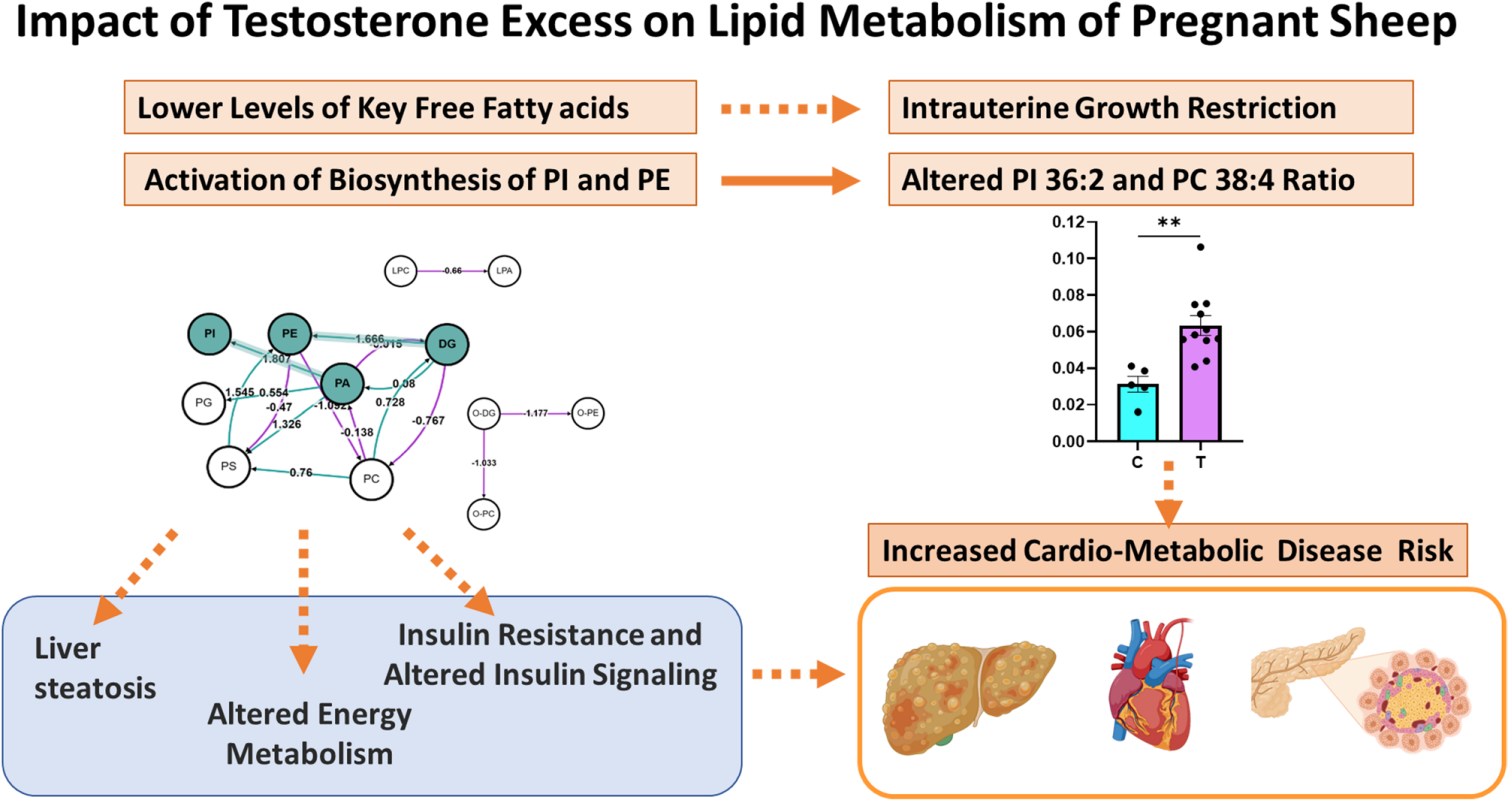
Schematic summary of T-treatment effects on maternal lipidome profile: T-treatment led to lower levels of key fatty acids and activated biosynthesis of PI and PE. Gestational T-treatment also resulted in higher PI 36:2 and 38:4 ratio, a feature reported to be related to increased cardiovascular disease risk. These perturbations in the lipid metabolism can be contributing factors to IUGR and adverse cardio-metabolic outcome evidenced in their offspring.

PI 36:2 and PC 38:4 ratio: Two of the lipid markers of T treatment with high VIP values were PI 36:2 and PC 38:4 (Fig. 4, top panel A) and PI 36:2 was also confirmed as a potential biomarker by S-plot for the T treatment (Fig. 4, bottom, panel B). PI 36:2 was upregulated in the T treatment whereas the PC 38:4 was downregulated in T group (Fig. 3, left panel A). The ratio of PI 36:2/PC 38:4 was significantly higher (*p* < 0.05) in the T group compared to the C group (Fig. 5, bar graph on the right).

## Discussion

Findings from this study expand upon our earlier findings of reduced acylcarnitines^48^ and addresses the adverse impact of T excess on the lipid metabolism in a precocial translationally relevant model for the development of PCOS phenotype in offspring. Our detailed profiling of maternal lipidome reveals that the major impact of the gestational T excess was exerted at the level of glycerophospholipids, glycerolipids and SMs. The key findings are (1) significantly lower levels of key fatty acids and acylcarnitines, and higher levels LNAPE at the class level and (2) activation of biosynthesis of phosphoinositides and phosphatidylethanolamine pathways. The schematic overview of the overall impact of excess T exposure on the maternal lipidome is summarized in Fig. 5.

The perturbations at the level of glycerophospholipids, glycerolipids, sphingolipids stemming from gestational testosterone treatment are similar to other pregnancy pathologies such as gestational diabetes (GDM)^51^ and preeclampsia^52,53^. The enrichment of glycerolipids and diglycerides at main and subclass level in the gestational T-treated sheep (this study) is also an aspect associated with GDM^54^. Similarly, sphingosine and ceramide, lipid subclasses that were enriched after T-treatment have also been reported to be enriched in preeclamptic pregnancies^55^. Of relevance to this study of gestational T excess, these pregnancy pathologies are also associated with higher androgen levels (GDM^56^; preeclampsia^57,58^). Other commonalities between gestational T-treated sheep and these pregnancy pathologies include maternal hyperinsulinemia (GDM^59^, preeclampsia^60,61^ and risk of intrauterine growth restriction (gestational T-treated sheep^62,63^; GDM^64^; preeclampsia^65^).

Maternal nutrient status and metabolism play key roles for proper growth and development of the fetus. Carnitines and acylcarnitines are fatty acid metabolites involved in many cellular energy metabolism pathways: fatty acid oxidation, energy metabolism, mitochondrial activity, insulin sensitivity, and oxidative stress^66^. The comprehensive lipidomics approach in addition to confirming our earlier findings of lower acylcarnitines at the class level^48^ has helped identify specific acylcarnitine markers that are likely contributors to intrauterine growth restriction and low birth weight evidenced in this model^62,67–69^. The disruptions in these metabolites may also be contributing factors to the hyperinsulinemic status evidenced in the gestational T-treated mothers^48^. Consistent with this premise, one study using targeted metabolomics of 400 pregnant women of European ancestry at 28 weeks of gestation found acylcarnitines to be associated with altered insulin sensitivity and newborn size^70^. This finding was later confirmed in a larger group of 800 pregnant women^71^. Specific acylcarnitine species found to be lower in maternal plasma in this study were also found to be lower in the offspring of gestational T treated lambs indicating long term effects on the fetal acylcarnitine metabolism exposed to excess T during pregnancy^72^.

Maternal fatty acids are important for placental development, feto-placental function and normal progression of pregnancy^73^. Lower levels of maternal fatty acids observed in the gestational T-treated mothers may have contributed to the placental insufficiency^62^ and compromised placental differentiation and function^62,74^ in this model. As key regulators of cell growth, cell signaling, and brain development, fatty acids are key regulators of fetal growth and development. A recent study reported increased levels of fatty acids (including saturated, monounsaturated and n-6 polyunsaturated acids) supportive of their higher requirement during pregnancy^75^. Of relevance to our findings, lower levels of fatty acids have been found to restrict fetal growth and development^75,76^. As such, the overall reduction in fatty acids levels evidenced in the gestational T-treated sheep likely limited supply of these key lipids to the developing fetus culminating in the IUGR and low birth weight offspring evidenced in this model^62,68^.

LNAPE and LPE: LNAPE and LPE, are lysoPE, which are formed after diacylation of PE by partial hydrolysis; although present in minute amounts in cell membranes, are important for cell signaling and lipid mediated responses^77–79^. There is limited information available about the role and function of these lipids in pregnancy. Our previous clinical study found LPE to be related to gestational weight gain and birth weight^80^.

PI biosynthesis pathway was found to be activated by gestational T excess*.* PIs are involved in protein–protein interactions, regulation of proteins, cholesterol transport and take part in key signaling pathways^81–83^. PI plays a role in fetal lung maturation and surfactant production^84,85^. PIs are key regulators of insulin signaling and energy metabolism^86^. The positive effects of inositides as insulin sensitizers^87,88^ suggest that activation of the PI biosynthesis pathway observed in this study could be a compensatory response to overcome the hyperinsulinemic status of gestational T-treated sheep, a risk factor for offspring health outcomes^89,90^.

Another pathway that was activated by gestational T excess was PE biosynthesis pathway*.* PE are one of the most abundant lipids classes^91^ and serve an important role in membrane permeability and fusion, mitochondrial function, oxidative phosphorylation and lipid transport. PE also serve as the substrate for biosynthesis of PC and the balance of PC and PE is important for liver function^92^. Activation of PE biosynthesis can lead to accumulation of PE and altered ratio of PE and PC, leading to membrane dysfunction, and ER stress^91,92^. Of interest, activation of PE biosynthesis pathway after T treatment during pregnancy may be one of the developmental programming effects of prenatal excess T as this pathway was also found to be affected in the newborn lambs after prenatal T treatment^72^.

Several key fatty acids were identified as potential biomarkers of gestational T excess**.** Polyunsaturated fatty acids (PUFAs) namely linoleic acid and arachidonic acid and the mono-unsaturated omega-9 fatty acid oleic, were identified as potential biomarkers of T exposure, with lower levels found in the T treated sheep. Arachidonic acid is important for proper functioning of cell membrane, immune and inflammatory systems and along with DHA is required for fetal brain development^76,93^. Linoleic acid is a precursor for biosynthesis of Arachidonic acid. As such, at times of linoleic acid deficiency, arachidonic acid becomes conditionally essential fatty acid^93^. Interestingly, PUFAs are reported to be lower in PCOS women^94,95^ a hyperandrogenic disorder the characteristics of which the female offspring of gestational T-treated sheep used in this study mimic^29–37^. While the mechanisms are unclear, several studies reported beneficial effects of PUFA supplementation in PCOS women^96,97^.

PI 36:2 and 38:4 were identified as the potential signatures of T excess during pregnancy in our sheep model. In addition to activation of the PI biosynthesis pathway, a higher ratio of PI 36:2 and 38:4 characterized the gestational T-treated sheep (Fig. 5). Lower PC 38:4 is likely the result of lower arachidonic acid and both PI 36:2 and 38:4 are linked to visceral fat metabolism^98^. These two lipids were reported recently as the lipid metabolism biomarkers for adverse cardiovascular outcome^99^. The same study also reported the negative association of arachidonic acid (also identified as potential biomarker of T excess in this study) and positive association of phosphoinositide species with adverse cardiovascular outcomes^99^. While we have not assessed the maternal cardiovascular consequences of gestational T treatment during pregnancy, we have found adverse sex-specific cardiac programming in their offspring^100,101^. Our preliminary findings also indicate adverse maternal cardiac remodeling during the postpartum period (Vyas, Padmanabhan, unpublished data). A review of literature indicates compromised maternal cardiac dysfunction in hyperandrogenic conditions^102^.

Strengths and limitations: The strengths of the study are the use of a well-established precocial model of translational relevance to hyperandrogenic disorders such as PCOS; pregnant sheep are highly sought out model to investigate maternal–fetal physiology^103^. Another strength is the use of the state-of-the-art plasma lipidome profiling that can be safely undertaken during pregnancy. There are several limitations to this study: (1) Although the blood sample for the lipidome analysis was procured during rapid growth phase of the fetus, the single time point estimate only provides a snap-shot view of lipid changes occurring at that time. Because lipid profiles are likely to vary at different gestational time points reflective of pregnancy-associated hormonal changes and consequent impact on lipid metabolism, the findings from this study while of interest only provides a snap-shot view that points to the need for undertaking more in-depth lipid profiling across multiple time points during gestation. (2) Because this was not set up as a feeding trial and the controls and testosterone-treated groups were housed and group-fed together, differences in feeding behavior, if any, cannot be accounted for. (3) The untargeted lipidome profiling used in this study, while offering a screening approach to assess relative abundances of various lipids, does not address causal relationships. (4) Levels of testosterone achieved in this sheep model are much higher than evidenced in human hyperandrogenic disorders. (5) There is potential for differences in lipid metabolism between ruminants and humans. Although digestion and absorption of dietary components will vary between ruminant and human and species differences in circulating concentrations of nutrients will exist, metabolism (i.e. the actual chemical reactions/metabolic pathways) are similar in mammals once carbohydrates, fats, proteins are broken down into glucose, lipids, and amino acids^104^. Of importance, ruminants are still glucose dependent except that they just require an extra step compared to non-ruminants (ruminal microbes convert carbohydrates to short chain fatty acids which is converted to glucose by the liver for utilization by target tissues). By the time lipids reach the small intestine, absorption into enterocytes and packaging is similar to that of non-ruminants^104^.

Future studies are needed to delineate the functional relationship of the lipids identified and their contributions throughout pregnancy and offspring outcomes as well as to address the potential contribution from changes in placental or fetal metabolism.

In summary, findings from this study provide evidence of the detrimental impact of gestational T excess in perturbing the maternal lipidome, a key component of maternal metabolism and fetal growth and development and hence a potential contributor to the IUGR and offspring cardiometabolic risk evidenced in this model (Fig. 5). The findings are of translational relevance to hyperandrogenic disorders such as PCOS, the characteristics of whom the offspring of gestational T-treated sheep mimic.

## Methods

**Figure 6:**
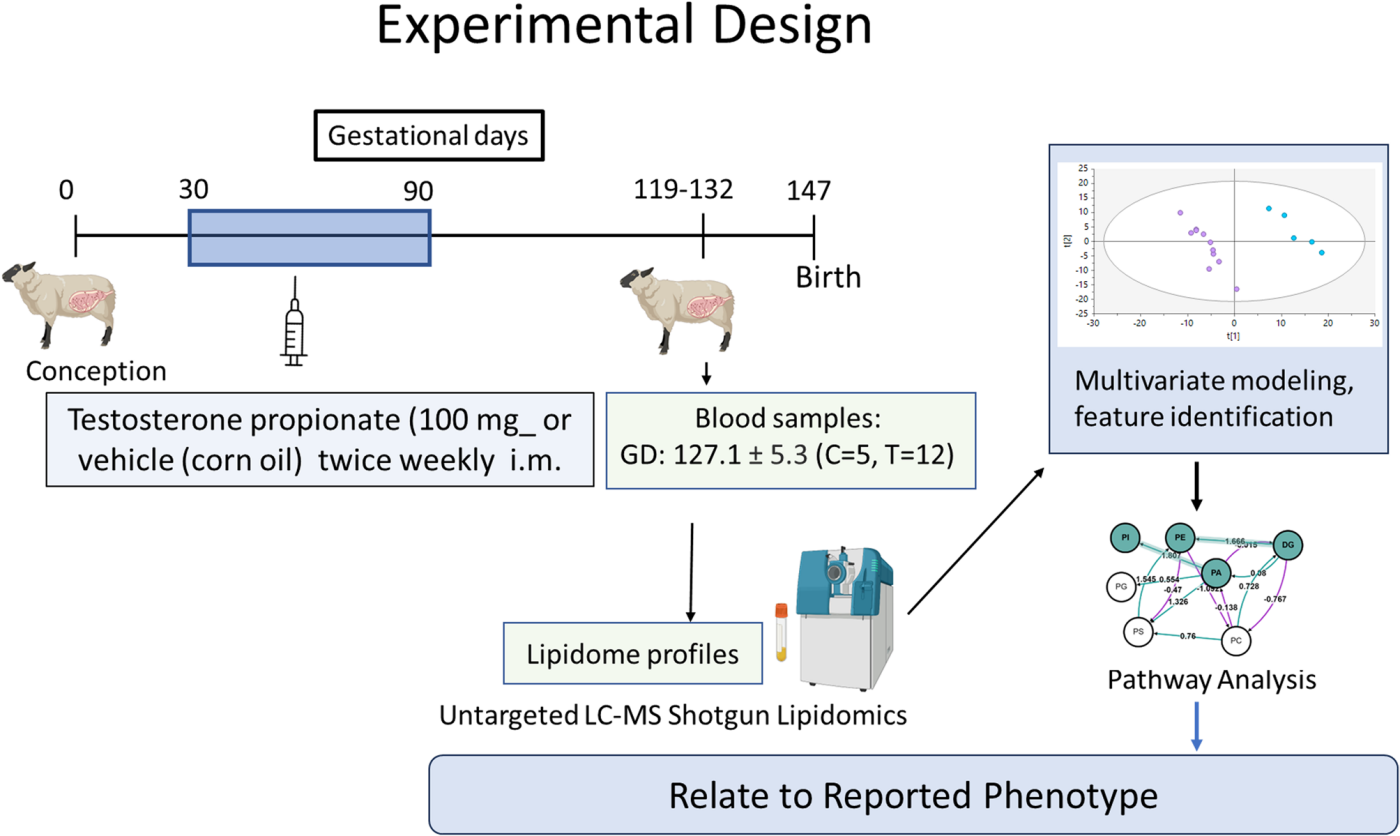
Experimental design: T propionate was injected subcutaneously twice weekly from days 30–90 of gestation. Plasma samples from ~ day 127 of gestation were subjected to Shotgun Lipidomics and data analyzed using multivariate dimensionality reduction modeling, feature identification and metabolic pathway analysis.

Study design (Fig. 6): All study procedures were performed in accordance with the National Research Council’s Guide for the Care and Use of Laboratory Animals and were approved by the University of Michigan Animal Care and Use Committee. Time-mated pregnant sheep were administered twice weekly injections of 100 mg testosterone (T) propionate (Sigma-Aldrich, St. Louis, MO) in 2 ml corn oil intramuscularly from days 30 to 90 of gestation (Term = 147days). Controls (C) were injected with 2 ml corn oil intramuscularly for the same duration in the same frequency. Pregnant ewes (control and testosterone-treated) were housed under a natural photoperiod and group-fed with a daily maintenance diet of 1.25 kg alfalfa/brome mix hay/ewe. To avoid transient effects of testosterone treatment, and focus on the rapid fetal growth period, blood samples (3 mL) were collected by jugular venipuncture at around day 127.12 ± 5.32 day (term 147 days) using BD vacutainer tubes (purple cap—EDTA tubes, Becton, Dickinson and Company, NJ), centrifuged at 3000g for 15 min, plasma separated and stored at -20°C until lipidomics analysis. Twice the number of breeder animals were assigned to the testosterone-treatment group in anticipation of considerable pregnancy loss, but most carried their pregnancy. Samples were collected from all available control and gestational testosterone-treated animals that were carrying their pregnancy, before their morning feed to avoid acute responses from food intake. This study was conducted and reported in accordance with ARRIVE guidelines^105^.

### Lipidome analysis

Shotgun Lipidomics was performed on plasma samples (N = 17, C = 5 and T = 12) at the University of Michigan Metabolomics Core. Plasma samples were spiked with internal standards and lipids were extracted using modified Bligh-Dyer method^106^ using a 2:2:2 ratio volume of methanol:water:dichloromethane at room temperature. The collected organic layer was dried under nitrogen. The dried extract of lipids was reconstituted in 100 μL of 10 mM ammonium acetate containing buffer (10:85:5 ACN/IPA/H_2_O). Data dependent Liquid chromatography-mass spectrometry (LC–MS/MS) was used to measure lipid species. Shimadzu CTO-20A Nexera X2 UHPLC systems (Canby, OR, USA). ABSCIEX 5600(AB Sciex, Concord, Canada) were used for chromatographic separation and triple Time of Flight Mass spectrometry (TOF MS) was used for Mass spectrometry. Both positive and negative ionization modes with a DuoSpray ion source (AB Sciex, Concord, Canada) were used for data acquisition to provide good separation of all the lipid classes. Lipid species were identified by data-dependent MS/MS product ion formation of plasma lipid species. Fatty acids composition was determined in the negative mode. After every 10 samples quality control (QC-pooled plasma) samples were injected at the beginning and end of each analysis. The raw data was converted to mgf data format using proteoWizard software^107^. The converted files were searched against LipidBlast library^108^ using NIST MS PepSearch Program libraries in batch mode^109,110^. Lipid species identified were from lipid classes PC, PE, PI, LPC, phosphatidylserines (PS), phosphatidylglycerols (PG), SM, Cer, triglycerides (TG), DG and monoglycerides (MG). Lipids were quantified using Multiquant and normalized by internal standards. Lipids detected in the study had 20.8% average coefficient of variation. All species within the lipid subclasses were combined and percentage of each class was calculated by relative abundance of each class against the sum of all lipid classes. Univariate Students’s *t*-test was used to calculate mean differences in lipid class analysis and raw *p* values and false discovery rate adjusted *p*-values (FDR adjusted) were calculated using Prism v10.1 (GraphPad, La Jolle, CA, USA) and MetaboAnalyst v5.0 (Canada).

### Dimensionality reduction modeling

Dimensionality reduction multivariate modeling was performed using SIMCA version 17 (Sartorius Stedim Data Analytics AB) to visualize the normalized lipidomics abundance data. Briefly, the normalized abundance data scaled using unit variance scaling (mean centered and divided by standard deviation). Unsupervised and supervised models were investigated for the clustering and patterns in the lipidomics data. Principal component analysis (PCA- unsupervised) was used for the overview and to identify patterns in the data for controls and gestational T-treated sheep. The model of first and second principal components explaining maximum variation in the data was used for the overview analysis, and two dimensional and 3 dimensional models were developed. Supervised models including partial least square discriminant analysis (PLS DA) and orthogonal partial least square discriminant analysis also known as orthogonal projections to latent structures (OPLS DA)^111^ were used to investigate C and T groupings and to identify potential biomarkers of T excess during pregnancy. To obtain information about grouping variables, PLS DA uses multivariate regression to obtain linear combinations of X variables (lipidomics data) with outcome Y variable (C and T groups). OPLS DA uses a technique where models are rotated to get the information about the outcome Y grouping variable in the first predictive component with all other variance explained in the orthogonal components^111,112^. Hotelling’s T2 tolerance ellipse (multivariate generalization of t distribution) was used to identify strong outliers. One outlier was detected in the PLS DA and OPLS DA models and the new multivariate models were developed after removing the outlier. All supervised models including PLS DA and OPLS DA models reported in this study had good model fit and predictability based on R2X and Q2 values indicating model fit and predictive ability, the supervised models used had R2Y (values above 0.95) and Q2 (values ≥ 0.45). The first two components of the PLS DA model and predictive component which represents data related to the outcome and the first orthogonal components for the OPLS DA were used to develop models. PLS-DA and OPLS-DA loadings and variable importance in projection (VIP) scores were used investigate lipid species responsible for the separation. Variables with loadings away from the center and with smaller confidence interval are responsible for the separation in the score plot. VIP values are weighted sum of squares of the PLS and OPLS loadings with the amount of Y-variation explained in each dimension. OPLS DA models provide information about the lipid species related to the outcome variable (in this case C and T groups) by separating unrelated information in the orthogonal component. These identified lipid species have the potential to serve as reliable biomarkers. Potential signatures of T excess during pregnancy were identified using VIP values and S-plots (OPLS DA). Lipid species with higher VIP values are important to the model and responsible for the separation of groups in the model. The S-plot provides information about the significant important lipid species by plotting model covariance against model correlations. The variables on the lower left and upper right corners have high significance and high magnitude change and these variables have the potential to serve as reliable biomarkers. Ratio of PI 36:2 and 38:4 was calculated using normalized abundancies and *t* statistics was used to test significance (*p* value < 0.05) in the difference between the C and T groups. Retrospective power analysis of the lipid signatures (identified by the OPLS DA S-plot) was performed using PS program (Vanderbilt University, Nashville, TN, USA).

### Enrichment analysis

Metabolite Set Enrichment Analysis (MSEA) was performed using MetPA, MetaboAnalyst v5.0 (Canada)^113^. MSEA identifies biological and functional patterns based on their significant enrichment in lipidome data. For the enrichment analysis, variables with VIP above 1, indicating their importance to the model, were selected. Enrichment analysis to identify lipid Superclass, Main classes, and Subclasses responsible for the separation of the C and T groups was performed. The list of lipid species with VIP above 1 was imported in the MetaboAnalyst enrichment analysis online tool and Over Representation Analysis (ORA) was performed. Overrepresentation analysis uses a hypergeometric test to estimate if certain Super classes, Main classes and/or Subclasses of lipids were represented more than expected by chance within the list of lipid species. The online tool uses compounds available in the MetaboAnalyst database and the lipid species which were not detected in the database were removed by the software and not included in the enrichment analysis. One tailed *p*-values were calculated after adjusting for multiple testing (false discovery rate adjusted *P*-value). MetaboAnalyst was also used to generate Heatmaps to investigate the top fifty lipid species based on Student’s *t*-test statistics.

### Pathway analysis

Lipid metabolism pathways were investigated using Bioinformatics Methodology for Pathway Analysis (BioPAN) on LIPID MAPS platform. For this analysis normalized lipid abundancies were imported to the online platform. Pathway analysis at the subclass and individual lipid species level were performed.

#### Differences in lipid metabolism pathways

To align the uploaded dataset with the BIOPAN nomenclature, the dataset was aligned to the software database by LipidLynxX. The lipid species were mapped in the uploaded lipidome data and lipid pathways were investigated to understand differences in C and T groups. A total of 738 lipid species were mapped by the software, of which 147 were found to be active in lipid metabolism reactions. For pathway analysis and lipid metabolism reactions *p* value of 0.05 was set as a threshold and lipid subclass and lipid species level analyses were performed. The pathway and reaction *z* scores were calculated and *z* score of 1.645 (*p* value 0.05) and above were considered significant. Pathways and reactions were investigated in reference to activation or suppression in the T-treatment group. Higher z scores indicated a shift towards more product of the pathway, which may lead to lower levels of the substrate reactants. Pathway active and suppressed reactions graphs with z scores were developed where green filled circles indicate significant pathways and reactions. Detailed perturbed pathways and reactions by testosterone excess with predicted genes involved in these pathways are represented in table format.

### Supplementary Information

Supplementary Tables.

## Data Availability

The supplementary data and the dataset generated and/or used for the analysis and interpretation of the results is available at the following link. Pease contact corresponding author (Vasantha Padmanabhan at vasantha@umich.edu) with any queries. https://figshare.com/s/4fbfe8cad4770b3311ae
