# Supplementary material for: Gestational testosterone excess early to mid-pregnancy disrupts maternal lipid homeostasis and activates biosynthesis of phosphoinositides and phosphatidylethanolamines in sheep
Source: Sci Rep. 2024 Mar 14;14:6230. doi: 10.1038/s41598-024-56886-6 (PMC10940674; doi:10.1038/s41598-024-56886-6)
Supplement: Supplementary file 1 — Supplementary Tables. [file 41598_2024_56886_MOESM1_ESM.pdf]

Supplementary Table 1: Top 50 Lipid species based on t-test.

Lipid Species	t.stat	P value	FDR adj P value
**FFA(22:0);[M-H]-**	5.8769	4.03E-05	0.015838
**FFA(18:2);[M-H]-**	5.8119	4.51E-05	0.015838
**Unknown_FAHFA 36:1**	5.7633	4.91E-05	0.015838
**FFA(18:1);[M-H]-**	5.5066	7.73E-05	0.01871
**Unknown_FAHFA 34:0**	5.2039	0.000134	0.021838
**CAR 16:0**	5.1969	0.000135	0.021838
**Unknown_FAHFA 38:4**	5.0471	0.000178	0.024652
**FFA(20:4) Arachidonic acid;[M-H]-**	4.8262	0.000269	0.032551
**Unknown_FAHFA 40:4**	4.7156	0.000331	0.035633
**Unknown_FAHFA 37:3**	4.6549	0.000372	0.035974
**FFA(20:1);[M-H]-**	4.4667	0.000532	0.046824
**FFA(24:1);[M-H]-**	4.3822	0.000626	0.050488
**CAR 18:0**	4.3362	0.000684	0.05093
**FFA(18:0);[M-H]-**	3.9186	0.001544	0.10677
**CAR 18:1**	3.8642	0.001719	0.10908
**Unknown_FAHFA 38:6**	3.8252	0.001856	0.10908
**PI 36:2**	-3.7438	0.00218	0.10908
**PC 40:5**	3.7369	0.00221	0.10908
**Unknown PC 40:5@7.38**	3.7369	0.00221	0.10908
**FFA(20:0);[M-H]-**	3.7267	0.002255	0.10908
**Unknown_FAHFA 36:4**	3.7023	0.002367	0.10908
**PC O-40:5**	3.5141	0.003437	0.13986
**CerP 42:2;2O**	3.4949	0.003571	0.13986
**Unknown_FAHFA 35:0**	3.4917	0.003594	0.13986
**FFA(16:0);[M-H]-**	3.4892	0.003612	0.13986
**SM 40:1;2O**	3.4429	0.00396	0.14745
**Unknown_FAHFA 32:3**	3.4	0.004313	0.15464
**PC 38:4**	3.3418	0.004842	0.16741
**FFA(24:0);[M-H]-**	3.2841	0.005432	0.18131
**PC 41:5**	3.2263	0.006093	0.1966
**Unknown_FAHFA 40:3**	3.1812	0.006665	0.20812
**PC O-39:5**	3.1525	0.007057	0.21346
**PE O-42:5**	3.1247	0.007457	0.21542
**Unknown PC 38:6@4.72**	3.1174	0.007566	0.21542
**FFA(22:2);[M-H]-**	3.0457	0.008724	0.23715
**PI 39:4**	-3.0354	0.008904	0.23715
**FFA(20:5);[M-H]-**	3.0264	0.009065	0.23715
**PC 36:4**	2.9505	0.010536	0.26415
**LPC O-24:1**	-2.9454	0.010643	0.26415
**TG 35:0**	-2.8642	0.012496	0.3024
**Unknown Unknown_FAHFA 38:4@1.74**	2.8173	0.013706	0.32274

**SM 43:2;3O**	-2.8064	0.014003	0.32274
**PE-Cer 34:2;2O**	2.7894	0.01448	0.32597
**PE O-40:6**	2.732	0.016207	0.35656
**SM 40:2;2O**	2.7202	0.016586	0.35679
**CAR 14:1**	2.6359	0.019559	0.38368
**CAR 20:0**	2.6306	0.019764	0.38368
**Unknown_FAHFA 35:2**	2.6172	0.020286	0.38368
**PC O-21:3**	-2.6125	0.020473	0.38368
**FFA(22:3);[M-H]-**	2.604	0.020816	0.38368

Supplementary Table 2: Lipid Species level active and suppressed lipid metabolism pathways.

**Active Pathways**	**Pathway classification**	**Z-score**	**Predicted genes**
DG(36:3)→PE(36:3)	Biosynthesis of PE (Glycerolipids and Glycerophospholipids), Biosynthesis of PC (Glycerolipids and Glycerophospholipids)	2.715	*CEPT1*
PE(30:0)→PC(30:0)	Biosynthesis of PC (Glycerolipids and Glycerophospholipids)	2.328	*PEMT*
DG(34:3)→PE(34:3)	Biosynthesis of PE (Glycerolipids and Glycerophospholipids), Biosynthesis of PC (Glycerolipids and Glycerophospholipids)	2.29	*CEPT1*
DG(32:1)→PC(32:1)	Biosynthesis of PS (Glycerolipids and Glycerophospholipids), Biosynthesis of PE (Glycerolipids and Glycerophospholipids), Biosynthesis of PC (Glycerolipids and Glycerophospholipids)	2.217	*CHPT1*
DG(38:6)→PE(38:6)	Biosynthesis of PE (Glycerolipids and Glycerophospholipids), Biosynthesis of PC (Glycerolipids and Glycerophospholipids)	2.148	*CEPT1*
DG(34:3)→PC(34:3)	Biosynthesis of PS (Glycerolipids and Glycerophospholipids), Biosynthesis of PE (Glycerolipids and Glycerophospholipids), Biosynthesis of PC (Glycerolipids and Glycerophospholipids)	2.114	*CHPT1*
DG(38:6)→PC(38:6)	Biosynthesis of PS (Glycerolipids and Glycerophospholipids), Biosynthesis of PE (Glycerolipids and Glycerophospholipids), Biosynthesis of PC (Glycerolipids and Glycerophospholipids)	2.062	*CHPT1*
DG(35:1)→PE(35:1)	Biosynthesis of PE (Glycerolipids and Glycerophospholipids), Biosynthesis of PC (Glycerolipids and Glycerophospholipids)	1.922	*CEPT1*
DG(35:2)→PE(35:2)	Biosynthesis of PE (Glycerolipids and Glycerophospholipids), Biosynthesis of PC (Glycerolipids and Glycerophospholipids)	1.904	*CEPT1*
PE(40:7)→PC(40:7)	Biosynthesis of PC (Glycerolipids and Glycerophospholipids)	1.858	*PEMT*
DG(32:1)→PE(32:1)	Biosynthesis of PE (Glycerolipids and Glycerophospholipids), Biosynthesis of PC (Glycerolipids and Glycerophospholipids)	1.851	*CEPT1*

DG(40:4)→PC(40:4)	Biosynthesis of PS (Glycerolipids and Glycerophospholipids), Biosynthesis of PE (Glycerolipids and Glycerophospholipids), Biosynthesis of PC (Glycerolipids and Glycerophospholipids)	1.829	*CHPT1*
PE(40:4)→PC(40:4)	Biosynthesis of PC (Glycerolipids and Glycerophospholipids)	1.822	*PEMT*
DG(36:1)→PE(36:1)	Biosynthesis of PE (Glycerolipids and Glycerophospholipids), Biosynthesis of PC (Glycerolipids and Glycerophospholipids)	1.793	*CEPT1*
DG(38:5)→PE(38:5)	Biosynthesis of PE (Glycerolipids and Glycerophospholipids), Biosynthesis of PC (Glycerolipids and Glycerophospholipids)	1.733	*CEPT1*
DG(34:2)→PE(34:2)	Biosynthesis of PE (Glycerolipids and Glycerophospholipids), Biosynthesis of PC (Glycerolipids and Glycerophospholipids)	1.678	*CEPT1*
DG(36:3)→PC(36:3)	Biosynthesis of PS (Glycerolipids and Glycerophospholipids), Biosynthesis of PE (Glycerolipids and Glycerophospholipids), Biosynthesis of PC (Glycerolipids and Glycerophospholipids)	1.65	*CHPT1*

Suppressed Pathways	Pathway Classification	Z-score	Predicted genes
PE(36:4)→PC(36:4)	Biosynthesis of PC (Glycerolipids and Glycerophospholipids)	1.768	*PEMT*
PE(40:5)→PC(40:5)	Biosynthesis of PC (Glycerolipids and Glycerophospholipids)	1.661	*PEMT*

Supplementary Table 3: Species level active lipid metabolism reactions.

**Active Reactions**	**Z-score**	**Predicted genes**
PA(36:2) → PI(36:2)	3.679	*CDS1*, *CDS2*, *CDIPT*
DG(36:3) → PE(36:3)	2.715	*CEPT1*
PE(30:0) → PC(30:0)	2.328	*PEMT*
DG(34:3) → PE(34:3)	2.29	*CEPT1*
DG(32:1) → PC(32:1)	2.217	*CHPT1*
DG(38:6) → PE(38:6)	2.148	*CEPT1*
DG(38:6) → PC(38:6)	2.062	*CHPT1*
PC(28:1) → DG(28:1)	2.045	No genes identified yet
DG(35:1) → PE(35:1)	1.922	*CEPT1*
DG(35:2) → PE(35:2)	1.904	*CEPT1*
PE(40:7) → PC(40:7)	1.858	*PEMT*
DG(32:1) → PE(32:1)	1.851	*CEPT1*
DG(40:4) → PC(40:4)	1.829	*CHPT1*
PC(40:5) → DG(40:5) → PE(40:5)	1.827	*CEPT1*
PE(40:4) → PC(40:4)	1.822	*PEMT*
DG(36:1) → PE(36:1)	1.793	*CEPT1*
DG(38:5) → PE(38:5)	1.733	*CEPT1*
DG(34:3) → PC(34:3) → PA(34:3)	1.709	*CHPT1*, *PLD1*, *PLD2*
DG(34:2) → PE(34:2)	1.678	*CEPT1*
DG(36:3) → PC(36:3)	1.65	*CHPT1*

Supplementary Table 4 : Species level suppressed lipid metabolism reactions.

**Suppressed Reactions**	**Z-score**	**Predicted genes**
PC(34:3) → DG(34:3)	2.294	No genes identified yet
PC(38:6) → DG(38:6)	1.866	No genes identified yet
PE(36:4) → PC(36:4)	1.768	*PEMT*
PE(40:5) → PC(40:5)	1.661	*PEMT*
